# Identification of miRNA in Sheep Intramuscular Fat and the Role of miR-193a-5p in Proliferation and Differentiation of 3T3-L1

**DOI:** 10.3389/fgene.2021.633295

**Published:** 2021-04-15

**Authors:** Fuhui Han, Lisheng Zhou, Le Zhao, Lei Wang, Lirong Liu, Haijuan Li, Jixian Qiu, Jianning He, Nan Liu

**Affiliations:** ^1^College of Animal Science and Technology, Qingdao Agricultural University, Qingdao, China; ^2^China Animal Health and Epidemiology Center, Qingdao, China; ^3^Aohan Fine Wool Sheep Stud Farm, Chifeng, China; ^4^Runlin Animal Industry Co., Ltd., Linqing, China

**Keywords:** sheep—lamb, RNA-sequencing, preadipocyte, proliferation and differentiation, target gene analysis, miR-193a-5p, *ACAA2*

## Abstract

Intramuscular fat (IMF) is one of the most critical parameters affecting meat quality and mainly affected by genetic factors. MicroRNA as an important regulatory factor, which is still a lack of research in the development of sheep IMF deposition. We used RNA sequencing (RNA-seq) and cell-level validation to explore the role of miRNA in IMF deposition. As for this purpose, longissimus thoracis et lumborum (LTL) samples of 2 month-old (Mth-2) and 12 months-old (Mth-12) Aohan fine-wool sheep (AFWS) were used to identified miRNAs expression. We found 59 differentially expressed miRNAs (DE-miRNA) between these age groups and predicted their 1,796 target genes. KEGG functional enrichment analysis revealed eight pathways involved in lipid metabolism-related processes, including fatty acid elongation and the AMPK signaling pathway. A highly expressed DE-miRNA, miR-193a-5p, was found to serve a function in 3T3-L1 preadipocyte differentiation. Luciferase assay demonstrated that miR-193a-5p directly binds to the 3′-UTR region of *ACAA2*. By constructing mimics and inhibitor vector transfecting into 3T3-L1 cells to explore the effect of miR-193a-5p on cell proliferation and differentiation, we demonstrated that overexpression of miR-193a-5p inhibited 3T3-L1 preadipocyte proliferation, as evidenced by decreased mRNA and protein expression of *CDK4* and *CyclinB*. CCK-8 assay showed that miR-193a-5p significantly inhibited cell proliferation. Similarly, the overexpression of miR-193a-5p inhibited 3T3-L1 preadipocyte differentiation and adipocyte-specific molecular markers’ expression, leading to a decrease in *PPARγ* and *C/EBPα* and *ACAA2*. Inhibition of miR-193a-5p had the opposite effects. Our study lists the miRNAs associated with intramuscular lipid deposition in sheep and their potential targets, striving to improve sheep meat quality.

## Introduction

The market demand and accepted quality of mutton are being higher with its nutritional value improvement in consumer awareness. At present, in the evaluation of the meat quality of mutton, the content of intramuscular fat (IMF) has become one of the important indexes to measure meat quality (Anderson et al., 2015). Indeed, IMF content is closely related to the flavor, juiciness, and tenderness of the meat (Dodson et al., 2015). Studies have shown that lamb IMF content must be maintained within the range of 4–5% or higher to satisfy the consumers’ flavor requirements (Mohrhauser et al., 2015). In the ongoing breeding process, the IMF content is generally low, resulting in serious negative effects on mutton tenderness and quality. Studies have shown that intramuscular lipid deposition is affected by multiple genes and signaling pathways, therefore, exploring the mechanism of fat deposition at the molecular level is an indispensable part of meat quality regulation. In order to explore the regulatory role of non-coding RNA (lncRNA, miRNA, circRNA) in the process of IMF deposition, we systematically identified the profiles of DE genes during intramuscular lipid deposition in sheep through high-throughput sequencing(Han et al., 2021).

MicroRNAs (miRNAs) are important post-transcriptional regulators of gene expression that play an important role in the early development of animals, cell proliferation and differentiation, cell apoptosis, oncogene expression inhibition, and fat metabolism (Tüfekci et al., 2014; Deb et al., 2018). Many miRNAs regulate adipocyte differentiation and deposition by targeting the PPARs, C/EBPs, and other transcription factor families (Shukla et al., 2011). miRNA role in lipid metabolism was first reported in Drosophila. Their loss of miR-14 caused cell apoptosis, phenotypic changes, and fat droplets increase in the adipocyte (Xu et al., 2003). miR-143 was the first miRNA found to regulate the differentiation of adipocytes. Its inhibitors also inhibit human adipocyte differentiation and down-regulate the expression of *PPAR72*, *FABP*, and *GLUT4* genes (Esau et al., 2004). Moreover, miR-143 promotes bovine fibroblast-like preadipocyte differentiation into mature adipocytes (Li et al., 2011). Kajimoto et al. (2006) screened and identified 80 miRNAs in mouse 3T3-L1 preadipocyte. Studies have shown that miR-27a inhibits the differentiation of sheep preadipocytes by specifically binding to *RXRα* 3′-UTR (Deng et al., 2020). The development of high-throughput sequencing technology has enabled an integrated analysis of the expression profiles of mRNAs and miRNAs involved in fat metabolism (Jiang et al., 2014; Zhang M. et al., 2019; Zhang Z. et al., 2019). These studies indicated that miRNAs have an important regulatory effect on fat metabolism.

There are many reports on studies looking into the role of miRNA in fat metabolism of mice (Kajimoto et al., 2006), humans (Li et al., 2011), pigs (Chen et al., 2019), cattle (Yang et al., 2017), and other animals (Sun et al., 2019). However, there are few reports on research related to fat metabolism in sheep. The available reports mostly focused on the subcutaneous fat (Kang et al., 2017), tail fat (Wang et al., 2014; Li et al., 2018), and visceral fat (Suárez-Vega et al., 2018). Research on intramuscular IMF is even rarer. Aohan fine-wool sheep (AFWS) is one of China’s unique meat-wool dual-purpose sheep breed. It is an excellent model for studying the role of miRNA in regulating IMF deposition (Liu et al., 2013). The purpose of our research was to identify differentially expressed (DE)-miRNAs during IMF deposition in sheep by high-throughput sequencing of LTL (Longissimus thoracis et lumborum) muscle samples. We aimed to explore the molecular mechanism through which miRNAs regulate the proliferation and differentiation of sheep IMF cells. The study provides a reference for sheep to select meat quality traits from the molecular level, and provides molecular breeding technology support for speeding up the breeding of meat-wool dual-purpose sheep.

## Materials and Methods

### Ethics Approval Statement

All experimental protocols and animals’ managements in the study were approved by the No. 5 Proclamation of the Ministry of Agriculture, China. The tissues were taken with informed consent of Qingdao Agricultural University (Qingdao, Shandong, China) and Qingdao AoTe Sheep Farm (Qingdao, Shandong, China).

### Sample Preparation

The sheep used in this study were raised at the AFWS Stud Farm (Chifeng, Inner Mongolia, China) and received the same feeding and management conditions as other sheep on the farm. A total of 12 healthy AFWS rams (3 individuals for each stage) at 2, 4, 6, and 12 months of age were used. Samples of the LTL muscle were collected for RNA extraction and IMF content determination. A part of sample was placed in RNAase-free Eppendorf tubes, snap-frozen in liquid nitrogen, and then stored at −80°C pending RNA extraction. The samples used for IMF content determination were chilled (with ice pack) and brought back to the laboratory. The sample collection method and protocol for the determination of IMF content were the same as in our previous research (Han et al., 2021).

### RNA Extraction, Library Construction, and Sequencing

Total RNA in the LTL muscle of Mth-2 and Mth-12 rams was extracted using TRIzol (Thermo Fisher Scientific, Waltham, MA, United States). The RNA extract purity was examined by a NanoDrop ND-2000 instrument (Thermo Fisher Scientific, MA, United States). RNA integrity and quantity were assessed by 1% agarose gel electrophoresis and Agilent Technologies 2100 Bioanalyzer (Agilent Technologies, Palo Alto, CA, United States). After a quality check, a small RNA library was constructed with 1 μg total RNA for each sample using TruSeq Small RNA Sample Prep Kits (Illumina, San Diego, CA, United States). Two libraries, including six samples, were subjected to single-end sequencing (1 × 50 bp) with Illumina Hiseq2000/2500 at LC-BIO (Hangzhou, Zhejiang, China).

### Sequencing Data Analysis and Identification of miRNAs

Raw data were provided in the FASTQ format. miRNA sequences were identified by ACGT101-miR v4.2 (LC Sciences, Houston, TX, United States). Reads were filtered out if they met any of the following exclusion criteria: (1) N% greater than 10% (N stands for bases that could not be determined); (2) 5′ adaptors contamination; (3) without 3′ adaptor; (4) missed insert; (5) contained poly-A, G, C, or T; (6) reads were shorter than 18 nt. We retained sequences whose length was 18–26 nt, and compared them for removing adapter dimers, junk, low complexity, common RNA families (rRNA, tRNA, snRNA, snoRNA) and repeats using Ensembl^1^, RFam^2^, and Repbase^3^ databases (Han et al., 2016). We performed length distribution statistics on the filtered valid data. Subsequently, the unique sequences of the 18–26 nt RNAs were mapped to the sheep precursor in miRBase 22.0^4^ using BLAST to identify known miRNAs. Length variations at the 3′ and 5′ ends and a mismatch within the sequence were allowed in the alignment. The mapped pre-miRNAs were further aligned against the sheep genome^5^ to determine their locations. The unmapped unique sequences were mapped to the sheep genome by BLAST using the RNAfold software^6^ to predict hairpin RNA structures containing sequences from the flank 80 nt sequences. RNAfold software^6^ was applied to predict the structures of novel miRNAs precursors, hairpins, secondary structures, and minimum free energy (Hu et al., 2012). The miRNA expression level was determined using a normalization method (Li et al., 2016).

### Identification of Differentially Expressed miRNAs and Function Analysis of Target Genes

DE-miRNAs were compared between the two age groups using the DE-Seq R package (1.8.3). The identified DE-miRNAs satisfied the following conditions: log2 (fold change) ≥ 1 or ≤ −1 and *P* < 0.01. The target genes of DE-miRNAs were predicted by TargetScan^7^ and miRanda^8^. We performed Gene Ontology^9^ enrichment analysis to annotate the biological processes involved in predicted target genes to better understand their functions. Based on the results, we perform the Wallenius’ non-central hypergeometric distribution (Mao et al., 2005). Target gene candidates were also subjected to the Kyoto Encyclopedia of Genes and Genomes^10^ analysis by KOBAS (Xie et al., 2011). GO (Gene Ontology) terms and KEGG (Kyoto Encyclopedia of Genes and Genomes) pathways were defined as significantly enriched when *P* < 0.05. The clustering map was drawn by the R package. A co-expression network was drawn with Cytoscape (Version 3.4.0) to clarify the functions of miRNA and target genes in regulating lipid metabolism. We further used the Pearson correlation coefficient to measure the linear correlation between miRNA and mRNA expression levels.

### 3T3-L1 Preadipocyte Differentiation and Oil Red O Staining

3T3-L1 cells were purchased from the China Infrastructure of Cell Line Resource (Beijing, China) and maintained in DMEM (Hyclone, Shanghai, China) supplemented with 10% fetal bovine serum (FBS; Gibco, Langley, OK, United States) in an incubator with 5% CO_2_ at 37°C. The culture medium was changed every second day. When confluence reached 70–80%, cells were detached by trypsin with 0.05% EDTA (Gibco, Carlsbad, CA, United States) and reseeded in new culture plates at a density of 5 × 10^6^ cells/plate. To induce differentiation to adipocytes, the preadipocytes were cultured in complete medium to a confluence of 70%, and then for two more days to reach contact inhibition. The culture medium was then replaced with MDI (methylisobutylxanthine, dexamethasone, insulin) induction medium (it is recorded as 0 d), a complete medium supplemented with 5 μg/mL bovine insulin (Solarbio, Beijing, China), 0.5 μmol/L 3-isobutyl-1-methylxanthine (Sigma, Shanghai, China), and 1 μmol/L dexamethasone (Sigma). The cells were cultured for 48 h in this medium, which was then replaced with insulin medium (DMEM + 10% FBS + 10 μg/mL insulin) to maintain differentiation for 48 h. Finally, the medium was replaced with a complete medium, and the cells were incubated until day 8. The adipocytes were harvested and stained by Oil Red O following published protocols (Ramírez-Zacarías et al., 1992).

### Dual-Luciferase Reporter Assay

*ACAA2* mRNA wild- and mutant-type 3′-UTR were amplified into the dual-luciferase reporter gene vector psi-CHECK2 (Promega, Beijing, China) between XhoI and NotI restriction enzyme cutting sites. The generated constructs were used to verify the target relationship between *ACAA2* and miR-193a-5p. These constructs were then co-transfected into 293T cells with miR-193a-5p mimics or negative control, and luciferase activity in the cells was detected by the Dual-Luciferase Reporter Assay System (Promega) 48 h later.

### Transfection of miRNAs Into Preadipocyte

3T3-L1 preadipocytes were seeded in six-well plates at a density of 5 × 10^4^ cells/well. When they reached a confluence of 70%, the cells were transfected using GP-transfect-Mate reagent (GenePharma Biotechnology, Shanghai, China). The miR-193-5p mimics, inhibitors, and negative controls (NC) were supplied by GenePharma. The concentrations of miRNA mimics, NC and inhibitor were 50 nM, following the manufacturer’s recommended range. After transfecting the cells for 48 h, they were collected for RNA and protein extraction. The remaining cells were cultured until they differentiated into mature adipocytes, following the above protocol. We evaluated the differentiation of mature adipocytes into lipid droplets by Oil Red O staining after transfecting the cells for 8 d.

### Cell Counting Kit-8 (CCK-8) Analysis

Cell Counting Kit-8 (CCK-8; Solarbio) analysis was performed following the manufacturer’s instructions. 3T3-L1 preadipocytes were plated in 96-well plates at a density of 1 × 10^4^ cells/well with 100 μL culture medium. When they reached a confluence of 60%, the cells were transfected with miR-193-5p mimics, inhibitors, and NC in five repetitions. After treatment for 24 h, 10 μL CCK-8 solution was added to the medium of each well and the plate was incubated for 4 h at which point absorbance was measured at 450 nm.

### Real-Time Quantitative PCR (RT-qPCR)

We randomly selected 10 miRNAs for quantification by the CFX96 Real-Time PCR Detection System (Bio-Rad, Hercules, CA, United States) to validate the mature miRNAs’ expression. Primers were designed using Primer 5^11^ and synthesized by Tsingke Biological Technology (Tsingke, Qingdao, China). Reverse transcription was conducted using 1 μg total RNA per sample, and the Mir-X miRNA First-Strand Synthesis Kit (TaKaRa, Dalian, China). *U6* and *GAPDH* were used to normalize the expression levels of mRNA and miRNAs, respectively. The sequences of the primers used are listed in Additional File 1 (Supplementary Table 1). The 20 μL PCR reaction system consisted of 10 μL SYBR^®^ Premix Ex Taq II (TaKaRa), 0.5 μL forward primer (10 μM/L), 0.5 μL reverse primer (10 μM/L), 1 μL cDNA, and 8 μL ddH_2_O. The following thermocycling program was used: 95°C for 10 min; 45 cycles of 95°C for 10 s, 60°C for 10 s, and 72°C for 10 s; 72°C for 6 min. Three independent replicates were conducted for each sample, and the relative expression levels were calculated using the 2^–ΔΔ*Ct*^ method (Livak and Schmittgen, 2001).

### Western Blot Analysis

The total proteins were extracted by RIPA Lysis Buffer (Beyotime,Shanghai,China), supplemented with phenylmethyl sulfonyl fluoride (Service-bio, Wuhan, China) at a ratio of 100:1 on ice. The extracted protein concentration was then measured. Proteins (30 μg) were separated by 10% SDS-PAGE polyacrylamide gel electrophoresis and then transferred onto PVDF membranes (Beyotime). The membranes were blocked with 5% skimmed milk for 2 h, and then incubated overnight at 4°C with rabbit anti-ACAA2, rabbit anti-PPARγ, rabbit anti-C/EBPα, rabbit anti-CDK4, and rabbit anti-CyclinB at a dilution of 1:2,000. The membranes were then incubated with an HRP-conjugated secondary antibody (1:2,000) for 1 h at room temperature, followed by washing. Rabbit anti-GAPDH was used as an internal control. All antibodies were purchased from Proteintech (Chicago, IL, United States). The protein signals were detected in a darkroom using Pierce ECL Western Blotting Substrate kit (Thermo Fisher Scientific, Dallas, TX, United States) according to the manufacturer’s instructions.

### Statistical Analysis

All the data from the RT-qPCR were obtained using at least three independent replicates. All charts were created using SPSS Statistics 17.0 (SPSS Inc., Chicago, IL, United States) and data represent the mean + S.E. Student’s *t*-test and one-way analyses were used to analyze the significance of differences between the groups (^∗^*P* < 0.05; ^∗∗^*P* < 0.01).

## Results

### Determination of IMF Content

The results of determination of IMF content have been published in previous studies (Han et al., 2021), and the results showed that the IMF content of 12 months of age was extremely significantly higher than that of 2 months of age, so Mth-2 (less lipid deposition) and Mth-12 (more lipid deposition) were selected for RNA sequencing (RNA-seq) to maximize the likelihood of detecting significant differences.

### Profiles of miRNAs Sequencing Data in Sheep Muscle Tissues

**FIGURE 1:**
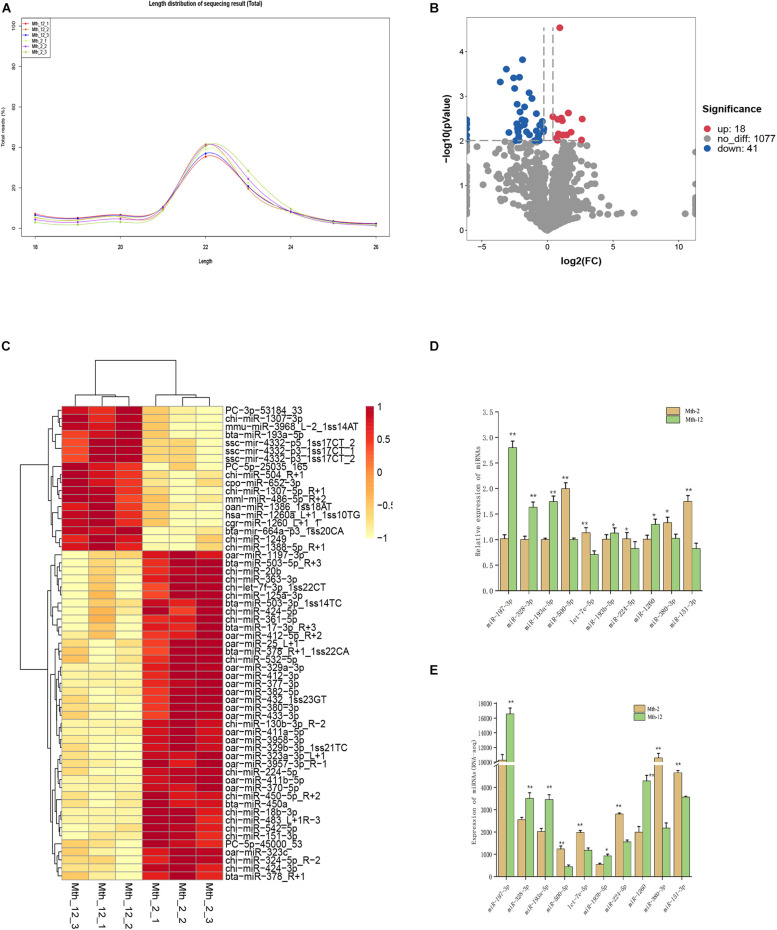
Characterization of miRNAs and Differentially expressed miRNAs analysis. **(A)** Length distribution of counts of total miRNAs. **(B)** The volcano of expressed miRNAs. The left blue points represent significantly down-regulated miRNAs; gray points represent miRNAs without significant changes; the right red points represent significantly up-regulated miRNAs. **(C)** The clustering maps of all the differentially expressed miRNAs. Red indicates that the gene had a higher expression level, and yellow indicates that the gene had a lower level of expression. **(D,E)** RT-qPCR validation of 10 differentially expressed miRNAs. U6 were selected as internal controls for miRNAs. ** Indicates that means were highly significantly different (*P* < 0.01); * indicates significant differences (*P* < 0.05).

We obtained LTL tissue samples from Mth-2 and Mth-12 rams for RNA-seq analysis, three replicates per sample, to study differential miRNAs expression related to lipid deposition. The average raw reads at Mth-2 and Mth-12 were 15,183,518 and 14,545,744, respectively, and the average numbers of valid reads were 11,141,632 (73.38%) and 8,836,507 (60.75%), respectively (Table 1). We retained reads with a length 18–26 nt and analyzed the length distribution of these miRNAs on the genome. The percentages of the 22-nt reads, the most abundant length (Figure 1A), were 39.16, 40.86, 40.53, 35.47, 41.45, and 36.98% for the six libraries. These results indicated that the reads could be used for subsequent analysis. The unique sequences of the 18–26 nt reads were mapped to the sheep precursor by BLAST to identify known miRNAs. A total of 1,247 miRNAs, including 1,136 unique ones, were detected. Of these, 983 conserved and 153 novel miRNAs were identified (Additional File 2: Supplementary Table 2).

**TABLE 1:** Summary of total RNA by RNA-sequencing.

Type	Mth-2		Mth-12	
	Counts	Percentage	Counts	Percentage
		(%)		(%)
Raw reads	15,183,518	100	14545744	100
3ADT and length filter	2,564,770	16.89	3627150	24.94
Junk reads	44,778	0.29	58298	0.4
Rfam	1,002,484	6.6	1654857	11.38
mRNA	359,021	2.36	220562	1.52
Repeats	70,833	0.47	148370	1.02
valid reads	11,141,632	73.38	8836507	60.75

### Identification of Differentially Expressed miRNAs in Sheep Muscle Tissues

We compared miRNAs between tissues from Mth-2 and Mth-12 rams using the DESeq R package (1.8.3), obtaining 59 DE-miRNAs, including 18 up-regulated and 41 down-regulated (3 novel and 56 known) miRNAs. The summary of the analysis results is shown as a volcano plot in Figure 1B, and a detailed DE-miRNAs list is presented in Additional File 3: Supplementary Table 3. Overall, the intramuscular fat (IMF) deposition mechanism in the Mth-2 and Mth-12 rams seemed highly related to the expression of these miRNAs. The clustering pattern of these 59 DE-miRNAs is shown in Figure 1C. The results of the RNA-seq are shown in Figure 1E. These results were consistent with those obtained by RT-qPCR for 10 randomly selected DE-miRNAs (Figure 1D), suggesting that the RNA-seq results were accurate.

### Prediction and Function Analysis of Target Genes

**FIGURE 2:**
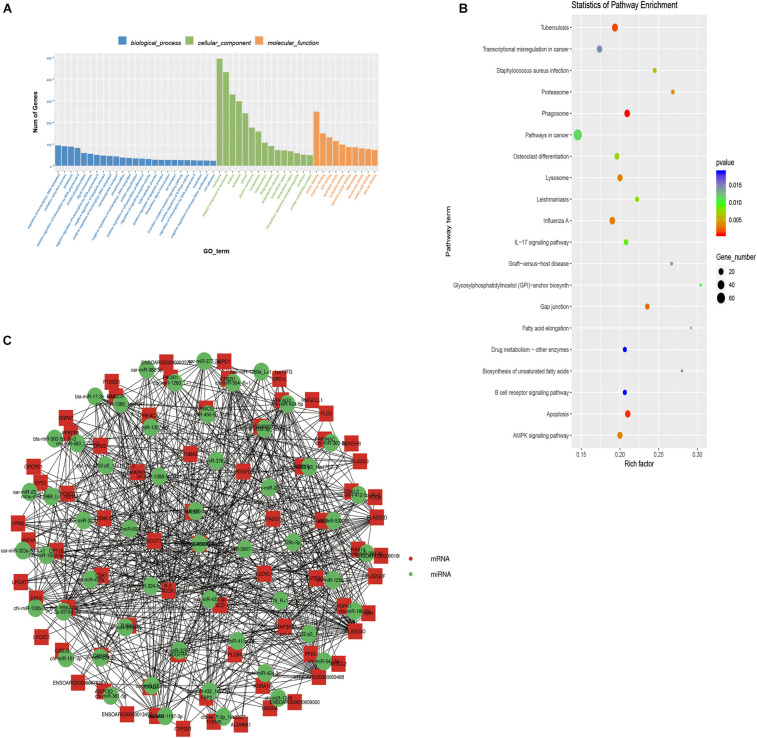
Function enrichment analysis of target genes. **(A)** The top 25 GO terms of biological processes, top 15 GO terms of cellular components, top 10 GO terms of molecular functions of target genes. **(B)** Top 20 KEGG pathways on targets of differentially expressed miRNAs. **(C)** Co-expression network for differentially expressed miRNAs and their targets. The red points represent critical mRNAs involved in lipid deposition. The green points represent candidate miRNAs, which can regulate target genes.

We used TargetScan and miRanda to predict the target genes of the 59 DE-miRNAs to determine their biological function. A total of 1,796 target genes were predicted for the DE-miRNAs (Additional File 4: Supplementary Table 4). We then performed GO and KEGG enrichment analyses to annotate the targets. The GO enrichment analysis results revealed that these target genes participated in a total of 433 significantly enriched functional classifications (*P* < 0.05). The biological processes group had the largest number of enriched genes (280 GO terms). This was followed by the molecular functions group with 93 GO terms and cellular components with 60 (Additional File 5: Supplementary Table 5A). Figure 2A shows the top 25 GO terms in biological processes, top 15 in cellular components, and top 10 in molecular functions. Among these, the most significant and highly enriched ones were peptidase activity (GO:0008233), proteolysis (GO:0006508), cytoplasm (GO:0005737), hydrolase activity (GO:0016787), endopeptidase activity (GO:0004175), Golgi apparatus (GO:0005794), cytosol (GO:0005829), and oxidation-reduction process (GO:0055114).

KEGG enrichment analysis can help identify the pathways these target genes participate in. Forty significantly enriched pathways (*P* < 0.05) were identified. The top 20 significant pathways are shown in Figure 2B. These include the AMPK signaling pathway (ko04152), gap junction (ko04540), and fatty acid elongation (ko00062; Additional File 5: Supplementary Table 5B). These pathways were shown to have a critical role in adipogenesis (Zhu et al., 2016; Coleman et al., 2019; Yao et al., 2019). The eight significantly enriched pathways related to lipid metabolism that we focused on (Table 2) included AMPK signaling pathway (ko04152), gap junction (ko04540), fatty acid elongation (ko00062), biosynthesis of unsaturated fatty acids (ko01040), alpha-linolenic acid metabolism (ko00592), glycerophospholipid metabolism (ko00564), linoleic acid metabolism (ko00591), and valine, leucine, and isoleucine degradation (ko00280). Of these, the fatty acid elongation pathway was significantly enriched by *ACAA2*, *HACD1*, *ELOVL7*, *ACOT7*, and more. Studies have shown that *ELOVL* and *HACD* gene families (Ikeda et al., 2008; Moon et al., 2009) catalyze the cycle-extending response of long-chain fatty acids. The *ACOT* gene family (Kang et al., 2012), including *ACOT7*, plays a key role in regulating hepatic lipid metabolism (Zhang et al., 2016). Additionally, *ACAA2* plays an important role in fatty acid metabolism by promoting sheep precursor adipocytes differentiation into adipocytes (Zhang et al., 2018). Based on the above research results, a network of 50 miRNAs and 23 mRNAs was established with Cytoscape. Among the DE-miRNAs, miR-193a-5p was noted to target the *ACAA2* gene (Figure 2C). It was inferred that miR-193a-5p might regulate lipid deposition by regulating the expression of *ACAA2*.

**TABLE 2:** Eight significantly enriched pathways related to lipid metabolism.

Pathway ID	Pathway term	Target gene list
ko04540	Gap junction	*PDGFD, TUBA8, MAPK3, CSNK1D, CDK1, TUBAL3, PDGFRA, PLCB4, MAP3K2, HTR2C, GJD2, ADCY9, ENSOARG00000010782, PRKCG, PRKACA, DRD2*
ko04152	AMPK signaling pathway	*FOXO3, CPT1B, PRKAA2, CAB39L, STRADA, FBP2, ADRA1A, PFKL, PDPK1, RAB14, CDKL3, SCD, PPP2R5C, PRKAG2, CREB1, PIK3R1, HMGCR, PFKFB1, GYS1, HNF4A, EEF2, CAB39, ADIPOQ*
ko00062	Fatty acid elongation	*ACAA2, ENSOARG00000005352, HACD1, ACOT7, THEM5, ELOVL7, HADHA*
ko01040	Biosynthesis of unsaturated fatty acids	*ENSOARG00000005352, HACD1, ACOT7, ACOX1, SCD, ELOVL7, FADS1*
ko00592	Alpha-Linolenic acid metabolism	*ENSOARG00000009488, PLA2G4D, PLA2G2F, PLA2G2D, ACOX1, PLA2G3*
ko00564	Glycerophospholipid metabolism	*PLD2, ENSOARG00000009488, GPD1L, GPD1, PLA2G4D, ENSOARG00000003375, PTDSS1, PLA2G2F, GNPAT, PLA2G2D, GPCPD1, LPIN2, LPGAT1, LPCAT2, ENSOARG00000007826, PLA2G3*
ko00591	Linoleic acid metabolism	*ENSOARG00000009488, ENSOARG00000013497, PLA2G4D, PLA2G2F, PLA2G2D, CYP2E1, PLA2G3*
ko00280	Valine, leucine, and isoleucine degradation	*ALDH6A1, ENSOARG00000009300, ACAA2, MCCC2, AUH, ENSOARG00000018083, PCCA, BCKDHB, HMGCLL1, HADHA*

### miR-193a-5p Binds to the 3′-UTR of *ACAA2*

**FIGURE 3:**
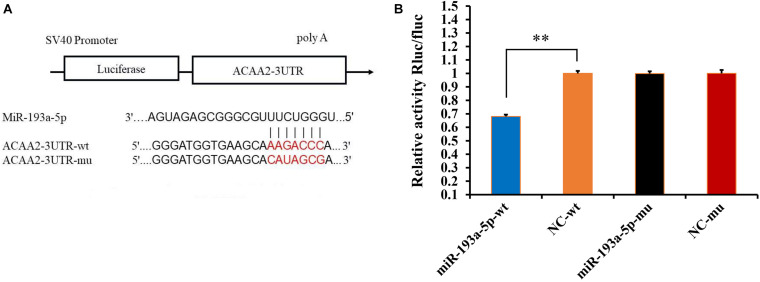
Verification of the targeting relationship between miR-193a-5p and *ACAA2* gene. **(A)** The predicted interaction of miR-193a-5p and *ACAA2*. **(B)** Verification of miR-193a-5p and *ACAA2* using dual-luciferase reporter assay. ** Indicates that means were highly significantly different (*P* < 0.01).

Based on the previous data analysis, we selected a highly significant differentially expressed miRNA, miR-193a-5p, to investigate its role in adipogenic differentiation. We determined, using TargetScan and miRanda, that miR-193a-5p had a seven-base potential binding site on the 3′-UTR of *ACAA2* (Figure 3A). We further verified their association through the dual luciferase system. The results, shown in Figure 3B, indicated that miR-193a-5p significantly down-regulated *ACAA2*-3′-UTR-WT luciferase expression (*P* < 0.001), suggesting that miR-193a-5p binds directly on the 3′-UTR region of the *ACAA2* gene and thus plays a role in regulating adipocyte differentiation.

### The Expression of miR-193a-5p in Preadipocyte Differentiation

**FIGURE 4:**
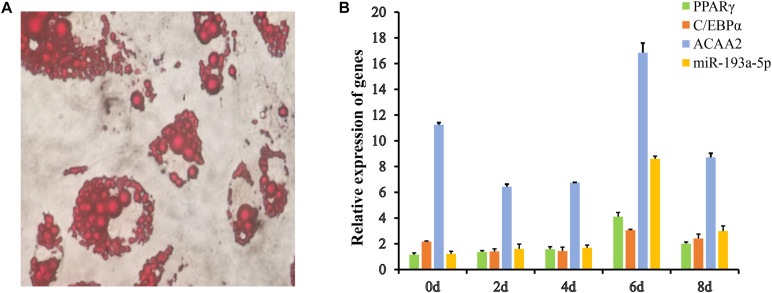
The Expression of miR-193a-5p, *ACAA2, PPARγ*, and *C/EBPα* in preadipocyte differentiation. **(A)** The Oil Red O staining results of pre-adipocytes. **(B)** The sequential expression of miR-193a-5p, *ACAA2*, *PPARγ*, and *C/EBPα* in 3T3-L1 preadipocyte differentiation.

The 3T3-L1 preadipocyte is a classic cell model for studying adipocyte proliferation and differentiation (Fan et al., 2019). We used it to study the role of miR-193a-5p in adipocyte development. The preadipocytes were induced and sampled 0, 2, 4, 6, and 8 d after in initiating differentiation, till they differentiated into mature adipocytes. The Oil Red O staining results of the preadipocytes on the eighth day after differentiation induction are shown in Figure 4A. Our results indicated that the preadipocytes were successfully induced to differentiate into mature adipocytes. We, respectively, determined the expression patterns of miR-193a-5p, *ACAA2*, and adipogenesis markers such as *C/EBPα* and *PPARγ*, in preadipocyte differentiation (Figure 4B). The expression of *ACAA2* showed a declining trend, then increase, and then decline again. The highest expression level was on day 6 after differentiation induction. The relative expression of miR-193a-5p increased gradually from day 2 to day 6 and then decreasing during differentiation. This expression result was similar in pattern to that of miR-125-5p, which was shown to inhibit the differentiation and proliferation of porcine intramuscular preadipocytes (Du et al., 2018). Therefore, we inferred that miR-193a-5p might inhibit preadipocytes differentiation.

### miR-193a-5p Inhibits 3T3-L1 Cell Proliferation and Differentiation

**FIGURE 5:**
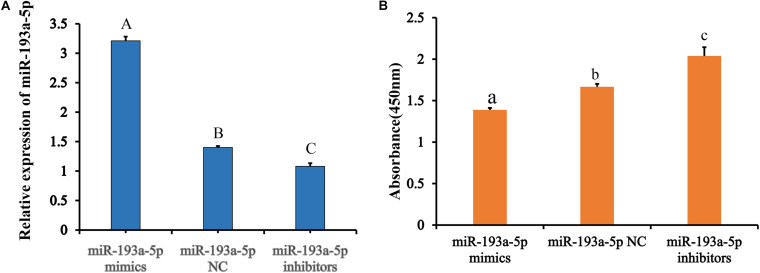
Transfection of miR-193a-5p vector and CCK-8 analysis. **(A)** Expression of miR-193a-5p after transfection of mimics and inhibitors. **(B)** CCK-8 analysis after treatment with miR-193a-5p mimic and inhibitor. Different lowercase letters indicate that means differ significantly (*P* < 0.05) and different capital letters indicate that means were highly significantly different (*P* < 0.01).

**FIGURE 6:**
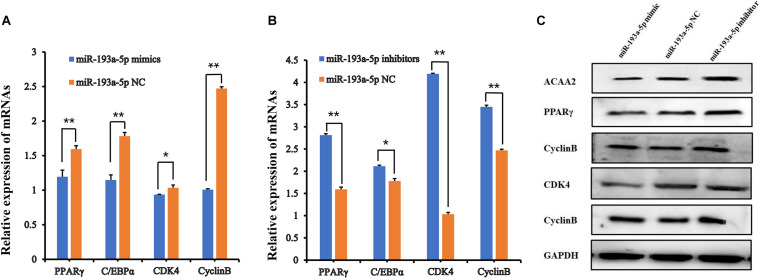
The functional assays of miR-193a-5p in 3T3-L1 preadipocyte proliferation and differentiation. **(A,B)** The expression of adipocyte-specific molecular markers (*PPARγ, C/EBPα*, *CDK4*, and *CyclinB*) by QPCR. ** indicates that means were highly significantly different (*P* < 0.01); * indicates significant differences (*P* < 0.05). **(C)** The protein expression of *ACAA2*, *PPARγ*, C/EBPα, *CDK4*, and *CyclinB*.

We transfected the preadipocytes with miR-193a-5p mimics, miR-193a-5p NC or miR-193a-5p inhibitors to further investigate the role of miR-193a-5p in preadipocytes proliferation and differentiation. The miR-193a-5p mimics significantly increased the amount of miR-193a-5p in preadipocytes, whereas the inhibitors reduced it (Figure 5A), showing that the chemical compounds were effective. We first explored the effect of miR-193a-5p on 3T3-L1 cell proliferation, using the CCK-8 assay. As shown in Figure 5B, 48 h after transfection, miR-193a-5p inhibition resulted in a significantly higher 3T3-L1 cell proliferation rate than in the miR-193a-5p NC group, while its overexpression significantly suppressed it. These findings indicated that miR-193a-5p might inhibit 3T3-L1 cell proliferation. Cyclin-dependent kinases (CDKs), such as cyclin-dependent kinases 4 (*CDK4*) and Cell cycle protein B (*Cyclin B*), have been recognized as essential for the progression of cell growth and proliferation in eukaryotes (Tsai et al., 1993; Ito, 2000). We determined expression level of *CDK4* and *Cyclin B* in cells transfected with miR-193a-5p mimics, inhibitors, or NC to further confirm miR-193a-5p-mediated inhibition of 3T3-L1 preadipocytes proliferation. RT-qPCR analysis illustrated that miR-193a-5p overexpression significantly down-regulated the expression levels of *CDK4* and *Cyclin B* (Figure 6A), while its inhibition strongly up-regulated their expression (Figure 6B). The CDK4 protein expression was inhibited by miR-193a-5p mimics and promoted by miR-193a-5p inhibitors (Figure 6C). The expression of *Cyclin B* was consistent with that of *CDK4* (Figures 6A–C). These results suggest that miR-193a-5p could inhibit 3T3-L1 cell proliferation.

**FIGURE 7:**
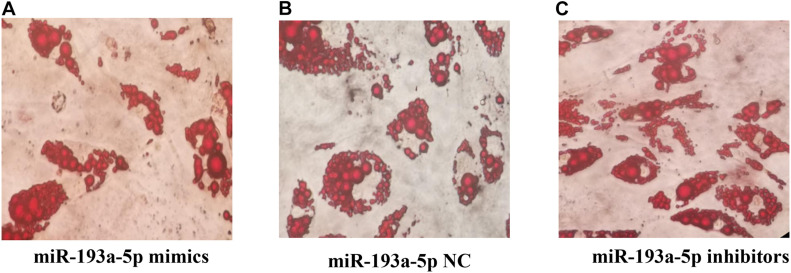
**(A)** The Oil Red O staining results after transfection of miR-193a-5p mimics. **(B)** The Oil Red O staining results after transfection of miR-193a-5p NC. **(C)** The Oil Red O staining results after transfection of miR-193a-5p inhibitors.

We further identified the functional role of miR-193a-5p during 3T3-L1 preadipocytes differentiation. By performing RT-qPCR analysis and western blot, we observed that miR-193a-5p mimics transfection for 48 h significantly suppressed the expression levels of adipogenic markers, including *C/EBPα* and *PPARγ*. Transfection with miR-193a-5p inhibitors remarkably promoted the expression of these factors compared to the miR-193a-5p NC group (Figures 6A–C). *ACAA2*, a key enzyme of the fatty acid oxidation pathway, playing an important role in fatty acid metabolism with catalyzing the last step of the mitochondrial beta-oxidation. RT-qPCR and western blot results indicated that miR-193a-5p mimics significantly suppressed *ACAA2* mRNA and protein expression, while miR-193a-5p inhibitors remarkably increased its expression (Figures 6A–C). These results suggested that miR-193a-5p might play a negative functional role during 3T3-L1 preadipocyte differentiation. The results of Oil Red O staining of 3T3-L1 preadipocyte on day 8 of differentiation also illustrated this point. The number of lipid droplets detected in differentiated cells in the miR-193a-5p mimics group was lower (Figure 7A) than the miR-193a-5p NC (Figure 7B), while it was higher after treatment with miR-193a-5p inhibitors (Figure 7C). These results demonstrated that by targeting *ACAA2*, miR-193a-5p inhibited 3T3-L1 preadipocyte differentiation.

## Discussion

Based on the results of the intramuscular fat (IMF) content determination, the IMF content of 12 months of age was extremely significantly higher than that of 2 months of age, so we selected 2 and 12 month-old samples to maximize the likelihood of detecting significant differences via RNA sequencing (RNA-seq). We initially identified 1,136 unique miRNAs in our study, including 983 conserved miRNAs and 153 novel miRNAs. Among them, there were 59 DE-miRNAs, including 18 up-regulated miRNAs and 41 down-regulated miRNAs. Studies have shown that intramuscular lipid deposition is directly related to the differentiation ability of intramuscular preadipocytes (Zappaterra et al., 2016). Some of the identified DE-miRNAs were previously shown to promote or inhibit preadipocyte differentiation into mature adipocytes. For instance, miR-125a-5p may promote 3T3-L1 preadipocyte proliferation but inhibit their differentiation by negatively regulating *STAT3* (Xu et al., 2018). miR-324-5p promotes adipocyte differentiation and lipid droplet accumulation by targeting Krueppel-like factor 3 (*KLF3*) (Zhou et al., 2020). miRNA-130b suppresses lipid deposition by negatively regulating target genes in pigs, cattle, and goats (Pan et al., 2014; Chen et al., 2015; Ma et al., 2018). miRNA-18b-3p, identified by RNA-seq, inhibits chicken intramuscular adipocyte differentiation by targeting *ACOT13* (Sun et al., 2019). Some miRNAs have also been identified as DE-miRNAs by previous RNA-seq studies related to lipid deposition, including miR-20b, miR-424-3p, miR-486-5p, and let-7f-3p (Hosui et al., 2017; Gasparotto et al., 2019; Huang et al., 2019; Ylioja et al., 2019). Interestingly, miR-664a and miR-542-5p were shown to play an important role in skeletal muscle development (Cai et al., 2018; Ji et al., 2020). Skeletal muscle development is inseparable from adipogenesis, so the regulatory mechanisms underlying these potential miRNAs require further study. These findings indicate that DE-miRNAs could potentially be regulators associated with adipogenesis.

GO enrichment and KEGG analysis were performed to investigate the biological processes associated with the DE-miRNAs target genes. We focused on eight significantly enriched pathways related to lipid metabolism (Table 2). Recent research found that these eight pathways participate in lipid metabolism and adipocyte differentiation (Habinowski and Witters, 2001). AMPK is one of the most important enzymes regulating energy metabolism in the cell, thus participating in the cellular metabolic process. Activating AMPK in 3T3-L1 cells can inhibit the expression of key adipogenesis genes, including *C/EBPβ*, *PPARγ*, and *C/EBPα* (Lee et al., 2012). Connexins are the protein building blocks of gap junctions, mediating cell-cell communication (Jiang and Gu, 2005). It was demonstrated that beige adipocytes display an increased cell-to-cell coupling via connexin 43 (Cx43) gap junction channels (Zhu et al., 2016). Fatty acid elongation pathway was significantly enriched by *ACAA2*, *HACD1*, *ELOVL7*, *ACOT7*, and others. These findings are similar to a previous study that found the *ELOVL*, *HACD*, and *ACOT* gene families to be significantly enriched in relation to fatty acid elongation (Ikeda et al., 2008; Moon et al., 2009). Metabolism of alpha-linolenic acid, glycerophospholipid, and linoleic acid are all lipid metabolism pathways. Studies have shown that these pathways are involved in the fatty acid composition of broilers and have an important regulatory effect on lipid deposition (Cui et al., 2020). Studies have also shown that various amino acids could be used to regulate intracellular signaling pathways, playing important biological roles in intracellular protein synthesis, transportation, and the body’s immunity (Logan and Nusse, 2004; Conner, 2016; Zeitz et al., 2019). Based on the above research results, we found that the target gene of miR-193a-5p, *ACAA2*, is involved in fatty acid elongation, and the glycerophospholipid and linoleic acid metabolism pathways. We inferred that miR-193a-5p is very likely involved in the differentiation of preadipocytes.

miRNA-193a-5p mostly focus on the research of human cancer in current reports and can be used as a diagnostic and therapeutic marker for many diseases. According to reports, its role is widely involved in the process of cell proliferation, differentiation, and apoptosis. miRNA-193a-5p can play a role through the regulatory network of circRNA-miRNA-mRNA or as a molecular sponge for lncRNA (Luo et al., 2019; Yang et al., 2019). Our study investigated the effects of miR-193a-5p on 3T3-L1 preadipocyte differentiation by transfecting the cells. We found that the relative expression of miR-193a-5p increased gradually from days 2 to 6 post-transfection and then decreased. This study investigated the effects of miR-193a-5p on 3T3-L1 preadipocyte differentiation by transfecting the cells. We found that the relative expression of miR-193a-5p increased gradually from days 2 to 6 post-transfection and then decreased. We inferred that miR-193a-5p is involved in the process of preadipocytes differentiation. Further functional assays proved that miR-193a-5p overexpression inhibited 3T3-L1 preadipocytes proliferation, as evidenced by the lower *CDK4* and *Cyclin B* mRNA and protein expression. CCK-8 assay showed that miR-193a-5p significantly inhibited cell proliferation. Similarly, overexpression of miR-193a-5p inhibited differentiation of 3T3-L1 preadipocytes and decreased the expression of adipocyte-specific molecular markers such as *PPARγ* and *C/EBPα*. Inhibition of miR-193a-5p had the opposite effects. The mRNA and protein expression levels of the target gene, *ACAA2*, were also inhibited by miR-193-5p. The *ACAA2* gene encodes an enzyme of the thiolase family, which participates in fatty acid metabolism by catalyzing the last step of the β-oxidation pathway (Miltiadou et al., 2017). Some research has shown *ACAA2* to affect meat quality, fatty acid metabolism, and lipid metabolism disorders (Broad and Ham, 1983; Yang et al., 2018). In summary, our research proved that miR-193a-5p inhibited the proliferation and differentiation of 3T3-L1 preadipocyte by targeting the *ACAA2* gene. Next, we will study the pathway mechanism of miR-193a-5p’s regulatory effect and further verify its regulatory effect at the individual level.

## Conclusion

Our study identified important miRNAs differentially expressed (DE) between two different phases (2 and 12 months-old) of development of AFWS. We also obtained the target genes and important key-pathways associated with preadipocyte proliferation and differentiation. We performed a complementary analysis to validate that a candidate miRNA, miR-193a-5p, inhibits preadipocyte proliferation and differentiation by targeting *ACAA2* gene. These findings lay the foundation for further explorations of the specific regulatory mechanisms of miRNAs on IMF deposition.

## Data Availability Statement

Additional data can be found in Supplementary Material. The RNA-Seq data was submitted to the SRA database under accession number SRR12247890.

## Ethics Statement

The animal study was reviewed and approved by the Qingdao Agricultural University Ethics Committee.

## Author Contributions

NL and JH designed this study. FH, LiZ, LeZ, and LW participated in sample collection. FH, LeZ, and LW performed RT-qPCR validation. FH, LiZ, NL, LL, HL, JQ, and JH analyzed the RNA-Seq data. FH, LiZ, LeZ, LL, and LW performed functional assays. FH wrote the manuscript with contribution from LiZ, NL, LeZ, LW, LL, HL, JQ, and JH. All authors reviewed and approved the final manuscript.

## Conflict of Interest

JQ was employed by company Runlin Animal Industry Co., Ltd. The remaining authors declare that the research was conducted in the absence of any commercial or financial relationships that could be construed as a potential conflict of interest.

## Footnotes

1 ftp://ftp.ensembl.org/pub/release-96/fasta/ovis_aries/

2 http://rfam.janelia.org

3 http://www.girinst.org/repbase

4 ftp://mirbase.org/pub/mirbase/CURRENT/

5 ftp://ftp.ensembl.org/pub/release96/fasta/ovis_aries/67

6 http://rna.tbi.univie.ac.at/cgibin/RNAWebSuite/RNAfold.cgi

7 http://www.targetscan.org/

8 http://www.microRNA.org

9 http://www.geneontology.org

10 http://www.kegg.jp/keg

11 http://www.premierbiosoft.com/index.html

## Supplementary Material

Supplementary Table 1 Primers used in the RT-qPCR analysis.

Supplementary Table 2 Summary of known and predicted miRNA in this study.

Supplementary Table 3 Summary of differentially expressed miRNAs.

Supplementary Table 4 The target mRNAs of the differentially expressed miRNAs.

Supplementary Table 5 **(A)** GO enrichment analysis of the differentially expressed miRNAs target genes. S gene number: the number of target genes which match to a GO term; TS gene number: the number of target genes which have GO annotations; B gene number: the number of detected target genes which match to a GO term; TB gene number: the number of all detected target genes which have GO annotations. **(B)** KEGG enrichment analysis (*P* < 0.05) of the differentially expressed miRNAs target genes.

## Abbreviations

AFWS: Aohan fine-wool sheep
IMF: Intramuscular fat
Mth-2: Mth-12: Rams at 2, 12 months
LTL: Longissimus thoracis et lumborum
RNA-seq: RNA sequencing
DE-miRNAs: Differentially expressed miRNAs
GO: Gene ontology
KEGG: Kyoto encyclopedia of genes and genomes
RT-qPCR: Quantitative real-time PCR
DMEM: Dulbecco’s Modified Eagle
*C/EBP α*: CCAAT/enhancer binding protein α
*PPAR γ*: peroxisome proliferator activated receptor γ
*CDK4*: cyclin-dependent kinases 4
*CyclinB*: Cell cycle protein B.
